# Propofol and Alfentanil in Treatment of a Patient With Episodic Cluster Headache

**DOI:** 10.5812/aapm.17560

**Published:** 2014-05-03

**Authors:** Sajjad Razavi, Babak Gharaei, Alireza Jafari, Homayoun Aghamohammadi, Alireza Mirkheshti

**Affiliations:** 1 Anesthesiology Research Center, Mofid Hospital, Shahid Beheshti University of Medical Sciences, Tehran, Iran; 2 Anesthesiology Research Center, Labbafinejad Hospital, Shahid Beheshti University of Medical Sciences, Tehran, Iran; 3 Anesthesiology Research Center, Imam Hossein Hospital, Shahid Beheshti University of Medical Sciences, Tehran, Iran

**Keywords:** Cluster Headache, Propofol, Alfentanyl

## Abstract

**Introduction:**

Cluster headache is a severe hemifacial pain with concomitant symptoms such as lacrimation, conjunctival congestion, and nasal discharge. Peripheral (to be a spectrum of trigeminal autonomic cephalgia) and central (hypothalamus) disorders have been suggested to be involved. Several modalities have been recommended to prevent or alleviate this devastating headache.

**Case Presentation:**

In this case report, we presented a young man with an acute cluster headache who responded dramatically to the treatment with propofol and alfentanil.

**Conclusions:**

Propofol and alfentanil combination can be considered as a treatment approach in the attack phase of cluster headache.

## 1. Introduction

Cluster headache is an intermittent hemifacial pain that is accompanied by autonomic dysfunction. Its prevalence ranges from 0.1% to 0.4% and mostly affects males (male to female ratio of about 5:1) with a mean age of 27 to 31 years at onset (1). Pain peaks at ten to15 minutes following the onset and usually lasts an hour (with a range of 15 to 180 minutes) (2).

Diagnostic criteria for the cluster headache include experiencing at least five attacks which accompany autonomic symptoms with a frequency of one every other day to eight episodes a day. Cluster headache has two types. In episodic type (85% of cluster headaches), attacks last from seven days to one year separated with pain-free period that lasts more than one month. In chronic form (15% of cases), patients experience nearly daily headaches throughout the year with no or short remission. Propofol shows promising response in treating refractory headache (3) while it was not effective in daily chronic headache. In the following case, we reported the application of propofol and alfentanil in successful treatment of a young man with episodic cluster headache.

## 2. Case Presentation

After taking written informed consent and informing the patient about possibility of reporting this case in a medical journal, a 45-year-old, 70 kg, 172 cm man with a history of episodic cluster headache was admitted to the hospital for pain management. He had periodic sharp excruciating pain on the left side of his face radiating from the back of his left eyeball. Lacrimation, conjunctival congestion, and nasal discharge were present on the left side. The present cluster was started one day ago. He experienced two severe episodes the day before; each lasted about 60 minutes with a six-hour pain relief between them. On the day of admission, he came with the same severe hemifacial pain initiated less than five minutes ago (he worked in the same hospital that researchers practiced). He was otherwise healthy and had no past medical history except some orthopaedic surgeries (due to an accident). He was not an alcohol or drug abuser. He had the same abrupt pain every three to six months that lasted about 15 days (the shortest and the longest clusters he remembered were five and 17 days, respectively). His symptoms started since five years ago and each year he experienced two to four clusters of headache on average, albeit he remembered a year with only a seven-day episode. The pain was not always completely improved with oral nonsteroidal anti-inflammatory drugs and very often, he was admitted to the hospital to receive oxygen therapy and intravenous painkillers. He mentioned that his first headache lasted six hours without any medication, as he was reluctant to receive any medication at first; however, he took painkillers for his following headaches. By taking medication, disabling episode of headaches lasted between 15 to 180 minutes; however, some mild to moderate pain remained for the same episode or following days of that cluster for which, he received trivial medication. He noted that he took just few hours sick leave and usually tolerated the pain. Once he received opioid (20 mg of intravenous meperidine) for his pain; however, it just added dizziness and nausea with no pain relief. He was reluctant to receive any prophylactic medication for his headaches. In his recent episode, he was admitted and hemodynamic and respiratory monitoring was utilized. Intravenous line was prepared and fluid was infused. He did not receive any supplemental oxygen. He received 20 mg of propofol (propofol 1% MCT/LCT, Fresenius, Germany) every four minutes (3) and simultaneous titrated dose of alfentanil (100 μg) every ten minutes. After the third injection of propofol and second prescription of alfentanil (about 15 minutes from the initiation of therapy), he reported acceptable pain relief with a mild remaining pain. He received a total dose of 120 mg of propofol (with the same protocol, however, as we encountered some drowsiness with no desaturation, we extended the interval for injection to every ten minutes and overall, it took about 50 minutes). Moreover, a total of 500 μg of alfentanil was infused (about 50 minutes). At this time he reported complete recovery with no remaining pain. He was just monitored and was discharged after two hours with no medication. He had no more episodes in the following days. In a four-month follow up, he did not experience any cluster headache.

## 3. Discussion

International Headache Society (IHS) defined the cluster headache as a severe unilateral, orbital, supraorbital, and/or temporal pain lasting 15 to 180 minutes untreated that is associated with at least one of the several signs ipsilateral with the pain, occurring in the attack frequency of one every other day to eight times a day (4). Considering the areas of brain involvement, there is great body of evidence indicating that cluster headache and trigeminal autonomic cephalgia are a spectrum of a headache syndrome (3). Different parts of central and peripheral nervous system including dopaminergic hypothalamic activity are supposed to be involved in the mentioned spectrum (5, 6). Several modalities have been used to treat or prevent acute attack of cluster headache including oxygen therapy, sumatriptan, lidocaine, ergotamine prescription, and high dose of methylprednisolone. Moreover, some new techniques such as sphenopalatine ganglion radiofrequency ablation or nerve stimulation (e.g. by rotigotine or sodium oxybate) have been applied to cease the pain in such cases (7-11). Propofol has been successfully used to treat migraine headaches (12-14). However, in a randomized trial, the single injection of propofol was not clinically effective in treatment of the chronic daily headaches (15). We did not find any documentation regarding propofol usage as a therapy for cluster headache. Several mechanisms have been proposed for the therapeutic effects of propofol on migraine headache. Suppression of central sensitization and spreading cortical depression are of theories that can be extrapolated to probable therapeutic effects in cluster headaches (16).

Opioid utilization in the treatment of headache is a controversial issue (17-20). Recent data support its application in the treatment of migraine headache (19, 20); however, caution should be taken regarding side effects such as the possibility of medication overuse and opioid-induced Hyperalgesia (18). In this case, considering ethical issues, we utilized a short acting opioid as an analgesic medication. To the best of our knowledge, this is the first report concerning combination of alfentanil and propofol as a therapeutic combination in a cluster headache patient. 

We chose half of the equianalgesic dose of alfentanil by considering data of opioid application in headaches (17, 19, 20). In this patient, we could not tell which treatment (propofol and/or alfentanil) had the utmost effect. However, patient's unsuccessful experiment (albeit with meperidine) with lone opioid might give us the clue that propofol had at least an adjuvant (if not the main) effect to control the headache.

In our case, the patient reported the best treatment he had ever received. Moreover, he noted that in previous therapies, he experienced a bothering remained pain following the relief of his pain attack. In conclusion, we can say that intravenous propofol and alfentanil terminated the acute attack of cluster headache and shortened the cluster episode of pain in this particular case. Further studies are required to determine the efficacy of this combination in cluster headache.
